# The Reward Positivity Tracks Positive Reward Prediction Errors From Feedback to Cues During Reinforcement Learning

**DOI:** 10.1111/psyp.70308

**Published:** 2026-05-12

**Authors:** Yifan Gao, Robert Wilson, Galit Karpov, Travis E. Baker

**Affiliations:** ^1^ Center for Molecular and Behavioral Neuroscience Rutgers University Newark New Jersey USA; ^2^ School of Psychology Georgia Tech Atlanta Georgia USA; ^3^ School of Medicine and Public Health University of Wisconsin Madison Wisconsin USA

**Keywords:** probabilistic selection task, reinforcement learning, reward positivity, reward prediction errors

## Abstract

How does the brain learn to predict rewards? According to temporal difference (TD) learning theory, reward prediction errors (RPEs) should shift from the time of outcome delivery to earlier predictive cues as stimulus‐action‐outcome associations are learned. The reward positivity, an electrophysiological signal believed to index sensitivity of the anterior midcingulate cortex to positive RPEs, should progressively transfer from feedback to predictive cues during learning. However, this core prediction of the reward positivity has remained largely untested. We recorded the EEG from 73 healthy adults performing a probabilistic selection task (PST) with extended training trials. The reward positivity amplitude was measured at both feedback and cue presentation during early and late training phases (first and second halves, respectively). To examine individual differences in RPE‐related learning processes, we split participants into rapid and slow learner groups and fit Q‐learning models to estimate separate learning rates for positive and negative feedback. Results showed clear evidence of temporal backpropagation: early in learning, the reward positivity appeared when feedback was delivered, but late in learning it disappeared from feedback and instead emerged when predictive cues appeared. Rapid learners showed more pronounced shifts in reward positivity from feedback to cue, consistent with their higher learning rates. These findings provide the first clear evidence that reward positivity demonstrates the temporal backpropagation predicted by TD learning theory. The results validate reward positivity as a neural marker of positive RPEs and highlight the importance of examining both cue‐related and feedback‐related brain responses to fully characterize reinforcement learning processes. Our findings have important implications for understanding individual differences in reinforcement learning and for interpreting the reward positivity in clinical populations and across the lifespan.

## Impact Statement

A widely held assumption is that the reward positivity reflects a positive reward prediction error (RPE), yet a core prediction of learning theory—that RPEs backpropagate from feedback to predictive cues—has never been formally tested. Our research provides the first clear evidence for this temporal backpropagation in humans. By showing that the reward positivity shifts from positive feedback to cues as participants learn stimulus–action–outcome associations, we validate it as a mechanistic marker of positive RPEs with implications for clinical biomarker development.

## 1 Introduction

The ability to use environmental cues to guide actions toward goals is a core component of reinforcement learning. Extensive empirical and computational work indicates that this fundamental mechanism is primarily driven by reward prediction error (RPE) signals—phasic bursts and dips in dopamine activity elicited when events are, respectively, ‘better than expected’ [positive reward prediction error (RPE)] and ‘worse than expected’ (negative RPE) (Schultz 2011, 1998). Specifically, RPEs reflect the difference between received and expected outcomes, being positive when events are better than expected and negative when they are worse than expected. In keeping with formal models of reinforcement learning, RPEs serve as a core component of temporal difference (TD) learning, a reinforcement learning algorithm that updates value estimates (also called state values—the expected future reward associated with each state) based on the difference between expected and actual rewards and values (Sutton and Barto 1998). A key feature of the TD algorithm is that RPEs transfer from the time of reward delivery to earlier predictive cues in trial‐and‐error learning tasks, progressively shifting to the earliest predictive indicator of reward. The backpropagation of RPE signals allows earlier cues and behaviors to acquire predictive value, enabling the learning of stimulus‐action‐outcome associations over time. To study this learning process in humans, researchers typically use probabilistic learning tasks in which participants make choices between options that provide probabilistic rewards (Sutton and Barto 1998). In these paradigms, participants are required to learn multiple concurrent discriminations between stimulus pairs (the cue), where one stimulus in each pair is associated with higher reward probability (e.g., 80%, 70%, or 60% of trials) while the other stimulus receives the complementary reward probability (e.g., 20%, 30%, or 40%) (Frank et al. 2004). According to TD learning theory, as participants learn through trial‐and‐error to choose the more frequently rewarded stimulus in each pair, positive RPEs should shift from unexpected reward delivery to the presentation of the stimulus pairs as those cues acquire predictive value, while RPEs to now‐expected feedback should diminish. This temporal migration allows earlier events in the learning sequence to acquire predictive value and guide future behavior.

Human event‐related brain potential (ERP) studies have sought to identify the neural signatures of these reinforcement learning processes (Holroyd and Coles 2002; Baker and Holroyd 2009; Cavanagh et al. 2010). In particular, when recording ERPs during probabilistic learning tasks, researchers identified a larger negative deflection (the N200 component) following negative feedback relative to positive feedback, peaking approximately 250 ms post‐feedback over frontal‐central regions of the scalp (Holroyd and Coles 2002; Miltner et al. 1997). This component is also observed in the frequency domain as frontal midline theta oscillations (FMT: 4–8 Hz) (Hajihosseini and Holroyd 2013; Cavanagh and Frank 2014). While this N200 component was initially interpreted as reflecting an error processing mechanism and termed the feedback error‐related negativity (fERN) (Miltner et al. 1997), subsequent work revealed that the difference between the ERPs elicited by feedback results mainly from a positive‐going deflection—the reward positivity—that is selectively elicited by unexpected positive feedback (Holroyd et al. 2008; Baker and Holroyd 2011; Cohen et al. 2007). Because the reward positivity and N200 overlaps both spatially and temporally on reward trials with opposing polarities, the N200 appears smaller, absent, or displaying more of a positive deflection in the ERP following positive compared to negative feedback, due to component overlap,^1^ not because of reduced error or conflict processing (Holroyd et al. 2008; Baker and Holroyd 2011). This observation led to the proposal that the N200 represents a default control response to unexpected task‐relevant events, while the reward positivity is produced by the impact of positive RPEs on the anterior midcingulate cortex (MCC), a cortical region that utilizes dopamine reward signals to learn the value of goal‐directed behaviors (Holroyd and Yeung 2012). Converging genetic, pharmacological, neuroimaging, and electrophysiological evidence supports the proposal that the reward positivity reflects positive RPE signals generated in the MCC, including modulation by dopaminergic genetic polymorphisms, selective attenuation by dopamine agonists, correlation with fMRI BOLD responses in the ventral striatum and MCC, and direct intracranial recording evidence identifying aMCC as the primary generator (Oerlemans et al. 2025; Baker, Stockwell, et al. 2016; Lau et al. 2026; Cavanagh and Holroyd 2026; Sambrook and Goslin 2015).

Despite extensive research on feedback‐related ERP components over the past three decades, a central prediction of TD learning theory remains largely untested: whether reward positivity amplitude reflects the backpropagation dynamics predicted by TD learning theory. While some studies have suggested this migration occurs (Baker and Holroyd 2009; Holroyd et al. 2011; Walsh and Anderson 2012), the evidence remains inconclusive due to both theoretical and methodological limitations. Theoretically, some researchers argue that feedback‐ and cue‐related reward positivity simply reflect enhanced salience responses rather than true RPE backpropagation. For example, Talmi et al. (2013) found that unexpected physical punishment elicited the reward positivity, arguing that the component reflects an unsigned salience prediction error for any motivationally significant outcome regardless of valence. Brown and Cavanagh (2018) similarly found enhanced P200 responses to predictive cues that disappeared when outcome uncertainty was introduced, supporting a salience rather than learning‐based interpretation. Methodologically, even studies reporting reward positivity to predictive cues have failed to test the dynamic learning process central to TD theory. Earlier investigations used either cues with fixed probabilities in passive viewing tasks or deterministic predictive cues with explicitly instructed contingencies (Baker and Holroyd 2009; Holroyd et al. 2011)—designs that eliminate the gradual learning process necessary for testing RPE backpropagation from outcome to cue. Additionally, subsequent ERP studies incorporating learning phases have focused exclusively on the fERN—the negative deflection in the ERP to negative feedback—rather than the reward positivity, the positive deflection in the ERP to positive feedback (Walsh and Anderson 2012), leaving it unclear whether observed ERP differences resulted from enhanced negativity to loss‐predictive cues or enhanced positivity to reward‐predictive cues. The present study addresses this limitation by measuring the N200 separately for positive and negative feedback conditions across learning phases, allowing us to directly dissociate learning‐related changes in the reward positivity (the positive deflection to positive feedback) from changes in the negative deflection to negative feedback, rather than collapsing both into a single difference waveform.

It is important to note that a key measurement consideration concerns the relationship between the reward positivity, the N200, and the fERN. Historically the fERN has been defined as the larger negative deflection in the N200 to negative compared to positive feedback and typically been measured using a difference wave (positive minus negative feedback ERPs) (Krigolson 2018; Proudfit 2015). However, as noted above, this apparent valence difference is not driven by an enhanced negative deflection to negative feedback, but rather by the presence of the reward positivity on positive feedback trials, which partially cancels the underlying N200. As we and others have argued, the fERN difference wave is therefore primarily driven by the presence or absence of the reward positivity rather than by modulation of the N200 to negative feedback per se (Holroyd et al. 2008; Baker and Holroyd 2011). Indeed, the difference wave approach has become the standard method for measuring the reward positivity, as it effectively eliminates neural activity common to both positive and negative feedback conditions—provided that feedback frequency is equal across conditions—thereby isolating the reward positivity as the positive‐going component uniquely elicited by positive feedback (Oerlemans et al. 2025; Krigolson 2018). However, the difference wave has important limitations for the present study (Krigolson 2018). First, it cannot determine whether learning‐related changes reflect increased positivity to positive feedback or decreased negativity to negative feedback, a distinction that is critical for testing RPE backpropagation. Second, in the PST, positive feedback occurs more frequently than negative feedback, meaning the difference wave is confounded by stimulus frequency effects (e.g., the oddball effect, whereby the N200 is enhanced to infrequent stimuli regardless of valence) making it impossible to cleanly isolate the reward positivity. Given the temporal and spatial overlap between the reward positivity and the N200 (Baker and Holroyd 2011), we therefore measured the reward positivity using condition‐specific N200 amplitudes separately for positive and negative feedback, and for predictive cues, across early and late learning phases. This approach allows us to directly track the reward positivity as a distinct positive‐going deflection in the N200 to positive feedback and test whether it transfers from feedback to cue as predicted by TD learning theory.

To test whether the reward positivity reflects RPE backpropagation, the present study examined two specific predictions of TD learning theory. First, reward positivity should systematically transfer from feedback to predictive cues as stimulus‐action‐outcome associations are acquired, showing a reciprocal relationship whereby cue‐related reward positivity increases as feedback‐related reward positivity decreases. Second, this temporal migration should correspond to individual differences in behavioral, electrophysiological, and computational measures of reinforcement learning efficiency. This individual differences approach is necessary for establishing the specificity of the reward positivity as an RPE signal: if temporal transfer of the reward positivity is genuinely driven by learning, then participants who acquire stimulus‐action‐outcome associations more rapidly, as indexed by behavioral accuracy and computational learning rate parameters, should show earlier and more complete transfer of the reward positivity from feedback to predictive cues relative to those who learn more slowly. To test these hypotheses, we used a modified probabilistic selection task (PST) (Biernacki et al. 2023), a standard trial‐and‐error learning paradigm for examining behavioral, computational, and electrophysiological mechanisms of human reinforcement learning (Frank et al. 2004, 2005; Cavanagh et al. 2010; Cavanagh and Frank 2014). To note, because its original design has been suboptimal for capturing the backpropagation of RPE‐related electrophysiological activity (see footnote^2^), we extended the training phase trial count and removed the training‐to‐test phase performance criteria. We believe these simple modifications to the task would enable a systematic examination of reward positivity at both cue presentation and feedback delivery as associations are acquired over time. Furthermore, to provide computational validation of learning, we fit Q‐learning models to individual participants' choice behavior (Sutton and Barto 1998; Watkins and Dayan 1992). Q‐learning estimates separate learning rates for positive (αGain) and negative (αLoss) RPE signals, allowing us to examine whether individual differences in reward positivity correspond to differences in computational learning parameters. Taken together, this study provides a definitive test of whether the reward positivity demonstrates the backpropagation of RPE signals or reflects alternative mechanisms such as salience processing.

## 2 Methods

### 2.1 Participant Recruitment and Study Procedures

A total of 80 undergraduate healthy participants participated in the study. Participants were recruited from Rutgers University—Newark Department of Psychology and the New Jersey Institute of Technology. Participants were compensated with either course credit or $25/h for their participation, in addition to a monetary bonus based on task performance. Participants were screened for current or previous neurological symptoms as well as history of neurological injuries (e.g., head trauma with loss of consciousness longer than 5 min) and asked to self‐report any psychiatric diagnoses. After the experiment, participants completed questionnaire measures. Following quality control of the EEG data, the final sample for analysis included 73 participants between 18 and 49 years old (Median = 20, Mean ± SD = 22 ± 6), with subjects self‐identifying Hispanic (*n* = 28), Asian (*n* = 13), Black (*n* = 10), White (*n* = 5), Middle Eastern (*n* = 9), and mixed raced (*n* = 8) individuals. This study was approved by the Institutional Review Board of Rutgers University, and all experiments were performed in accordance with relevant guidelines and regulations. The study adhered to the principles expressed in the 1964 Declaration of Helsinki. Informed consent was obtained from all participants.

### 2.2 Probabilistic Selection Task (PST)

**FIGURE 1:**
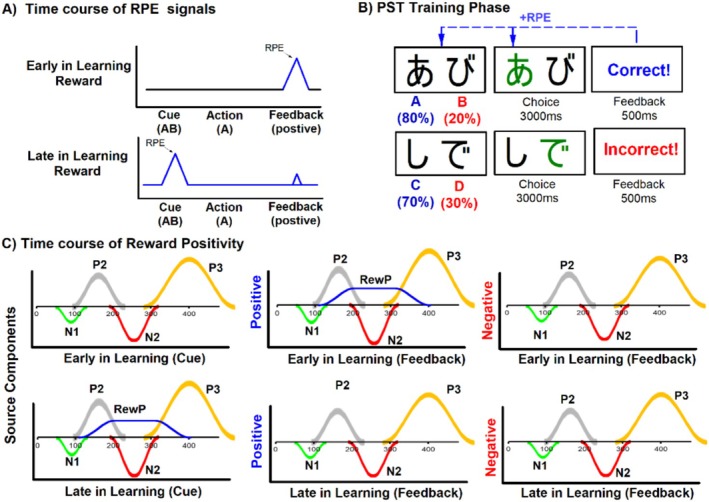
A schematic representation of the hypothesized time course of reward prediction error (RPE) signals and reward positivity during the PST. (A) The temporal difference (TD) model describes the time course of RPE signals early (top panel) and late (bottom panel) in learning during the training phase of the PST (B). RPEs transfer from the feedback (Positive Feedback) to the stimulus pairs (AB) as value gets updated. (C) Panels show two different sets of underlying source components for cue (left panel) and feedback (middle and right panels) that sum together to produce the observed ERP waveform. A complication that arises when the underlying components reward positivity and N2 sum together to form the observed ERP waveform, which creates an apparent reduction in N2 amplitude to positive feedback early in learning, and reduced N200 amplitude to cues late in learning.

Participants completed an adapted version of the PST (Figure 1B). The original PST includes a third pairing (EF, rewarded 60% and 40% respectively), for which participant accuracy is often at or near chance. To make the task easier for subjects to learn, and to maximize trial count for AB and CD pairs, we removed the EF pair from the stimulus set. In addition, we also removed the train‐test phase performance criteria commonly used. Thus, the PST consisted of four blocks of sixty trials each (240 trials total), followed by a subsequent testing phase. During the training phase, the participants were presented with two stimulus pairs, where each stimulus was associated with a different probabilistic chance of receiving “Correct” or “Incorrect” feedback. These stimulus pairs (and their reward probabilities) were termed A/B (80%/20%) and C/D (70%/30%). Over the course of the training phase, a participant usually learns to choose A over B, and C over D, solely due to adaptive responding based on the feedback. Following 240 trials, participants advanced to the testing phase. During the testing phase, participants were exposed to all possible combinations of these stimuli (i.e., AB, CD, AC, AD, BC, BD) in a random order and were required to select the symbol in each pair that they believed to be correct, but without receiving any feedback about their choices. Each test pair was presented four times.

### 2.3 PST Behavioral and Computational Analysis

Consistent with standard practice, we analyzed overall PST training phase accuracy and reaction time for AB and CD stimulus pair across Half‐1 [Block 1 and 2] and Half‐2 [Block 1 and 2]. For the test phase, accuracy for ‘approach learning’ was scored as selecting the most‐often rewarded A stimulus when paired with stimuli C or D, and for ‘avoidance learning’ as not selecting (avoiding) the least‐often rewarded B stimulus when paired with C or D. We then fit the training phase choice data using Q‐learning model, a reinforcement learning model that provides a means to simulate individual trial‐to‐trial task choices to determine separate learning rates for positive and negative RPE signals (Biernacki et al. 2023). The gain (αG) and loss (αL) learning rates determine the degree to which recent RPEs affect the expected value (Sutton and Barto 1998; Watkins and Dayan 1992). In brief, this model assigns expected reward values to actions taken during a particular state (i.e., choosing action A when seeing an A/B stimulus pair). These state‐action values are referred to as Q values. The model used separate learning rate parameters for gain (correct, αG) and loss (incorrect, αL) feedback trials in the training phase of the PST, and these separate learning rates scaled the updating of the Q values separately for rewards and punishments. Briefly, on each trial, a reward prediction error (RPE) is computed as the difference between the reward received and the current expected value of the stimulus: RPE = *R*(*t*) − Q*i*(*t*), where R(*t*) is the reward received at time *t* (1 for correct, 0 for incorrect) and Q*i*(t) is the expected value of stimulus *i*. Positive RPEs occur when outcomes are better than expected, and negative RPEs when outcomes are worse than expected. The Q value is then updated as the product of the learning rate and the RPE, such that the expected value (Q) of any stimulus (*i*) at time (*t*) was updated after each reinforcer:

$$Qi\left(t+1\right)=Qi\left(t\right)+\mathrm{\alpha G}{\left[R\left(t\right)-Qi\left(t\right)\right]}_{+}+\mathrm{\alpha L}{\left[R\left(t\right)-Qi\left(t\right)\right]}_{-}$$

where the learning rate applied is either αG or αL depending on whether the outcome is better or worse than expected, respectively. These *Q* values were entered into a softmax function to produce response probabilities (P) for each trial, with a free parameter for inverse gain (*β*) reflecting the tendency to explore or exploit:

$$Pi\left(t\right)=\exp\left(Qi\left(t\right)/\beta \right)/\Sigma j\;\exp\left(Qj\left(t\right)/\beta \right)$$

The parameters that maximized the log likelihood of the observed choices were identified using the Simplex method (fmincon, MATLAB), providing individual model fits characterized by pseudo‐R^2^ statistics ([LLE − *r*]/*r*, where *r* is the log likelihood of a chance model). All Q‐learning methods can be found here (Cavanagh et al. 2010; Biernacki et al. 2023).

### 2.4 Data Acquisition and Analysis

EEG was recorded using a 32‐channel actiCAP snap active electrode system, in conjunction with the actiCAP control box and the QuickAmp (Brain Products GmbH, Munich, Germany). The EEG was sampled at 1000 Hz and recorded to disk using Brainvision Recorder Software. The ground was positioned at channel AFz, and the designated online reference channel was set to channel Oz, which were connected to the actiCAP control box. To note, the QuickAmp uses an average reference for online recording, and the acti‐cap electrode system requires a designated reference channel at the time of acquisition and impedance check. Because the signal at this designated reference site is consumed during online referencing, it cannot be recovered during offline analyses; consequently, channel Oz was absent from the final ERP dataset. All subsequent analyses were therefore conducted on data offline re‐referenced to the bilateral mastoids.

EEG signals were processed offline using Brain Vision Analyzer 2 software. The data were subject to zero‐phase shift Butterworth infinite impulse response (IIR) filtering with low and high cutoffs set at 0.1 and 20 Hz respectively, and a 60 Hz notch filter. Filtered signals were re‐referenced offline to the bilateral mastoids. Manual rejection of noisy segments was performed before the ICA to correct for ocular artifact. A horizontal eye channel (hEOG) was created by subtracting channel FT9 from FT10. Channel Fp2 was designated for vertical eye activity (vEOG). These eye channels were used for the automated ocular ICA algorithm. ICA parameters included a Mean Slope Algorithm, with vEOG and hEOG channels referenced to a common reference using continuous data and Extended Infomax ICA. The convergence bound was set to 1E‐07, and the number of ICA steps to 512. ICA components were found using Sum of Squared Correlations with vEOG and hEOG, with 30% of the variance captured by the component deleted.

After ocular correction, data was segmented into 800 ms epochs time‐locked to cue and feedback, spanning from −200 ms pre‐stimulus to 600 ms post‐feedback. The data was then baseline corrected using the average activity from −200 to 0 ms. Artifacts rejection was based on a ±100 μV maximal allowed amplitude and a 50 μV voltage step as rejection criteria on the following 28 channels: C3, C4, CP1, CP2, CP5, CP6, Cz, F3, F4, F7, F8, FC1, FC2, FC5, FC6, FP1, Fz, O1, O2, P3, P4, P7, P8, Pz, T7, T8, LM, and RM. After artifact rejection, channels with artifacts exceeding 10% were interpolated using the signals from their four nearest neighbors (Hjorth 1975; Lin et al. 2022). Finally, the segments were divided into and averaged for Cue (first half, second half, first half, second half) and feedback (positive first half, positive second half, negative first half, negative second half). Overall, the data quality was excellent across the sample. Artifact rejection was minimal, with less than 0.5% of cue and feedback segments rejected in total. Only 5 of 73 participants required channel interpolation using the Hjorth nearest‐neighbor method, with a maximum of 2 channels interpolated for any single participant, and all participants retained a minimum of 28 channels for analysis. The mean number of retained trials per participant across conditions was as follows—cue‐locked: Half‐1 (*M* = 110.8, SD = 10.4), Half‐2 (*M* = 110.4, SD = 11.9); positive feedback: Half‐1 (*M* = 70.7, SD = 13.6), Half‐2 (*M* = 73.1, SD = 14.1); negative feedback: Half‐1 (*M* = 40.2, SD = 10.4), Half‐2 (*M* = 36.4, SD = 11.1). Cue and feedback trial counts reflect AB and CD stimulus pairs combined.

### 2.5 Reward Positivity (RewP)

While the reward positivity is typically measured using a difference wave (positive minus negative feedback ERPs) (Krigolson 2018), this approach cannot determine whether learning‐related changes reflect increased positivity or decreased negativity in the ERP waveform. Given the temporal and spatial overlap between reward positivity and the N200 component (Baker and Holroyd 2011), we measured the reward positivity using the condition specific N200. By measuring reward positivity amplitude separately for each stimulus type (cue vs. feedback) and learning phase (early vs. late), we tested the specific temporal backpropagation dynamics predicted by TD learning theory—namely, that reward positivity should diminish at feedback and emerge at predictive cues as learning progresses (see Figure 1). More specifically, the reward positivity to positive outcomes should decrease as rewards become expected, thereby exposing the N200 and making the waveform more negative in the second half. At cue presentation, the reward positivity should emerge as cues acquire predictive value, overlapping with the N200 and making the waveform more positive in the second half.

We tested these predictions by comparing N200 amplitudes at both feedback and cue presentation across training phase halves. In addition, because PST stimulus probabilities create confounds between stimulus frequency (e.g., P300 Oddball effect) and valence (Krigolson 2018), we examined the broader post‐stimulus waveform to assess other ERP components (P200, P300) that may overlap with or be modulated by reward positivity, providing supporting evidence for component dissociation and learning‐related changes. Thus, the reward positivity (as measured using the N200), P200, and P300 mean amplitude were measured within a window of ±25 ms centered around the corresponding cue and feedback averaged peak latency.

Cue‐related ERPs were measured as follows—P200 peak latency (Fz): 170 ms, window: 145–195 ms; Reward Positivity: N200 peak latency (Fz): 285 ms, window: 260–310 ms; P300 peak latency (Pz): 490 ms, window: 465–515 ms. Feedback‐related ERPs were measured as follows—P200 peak latency (Fz): 210 ms, window: 185–235 ms; Reward Positivity: N200 peak latency (Fz): 270 ms, window: 245–295 ms; P300 peak latency (Pz): 360 ms, window: 335–385 ms. To note, this approach directly captures reward positivity activity during learning while accounting for genuine amplitude and latency differences between cue‐related and feedback‐related ERPs. For example, the N200 peaked at 285 ms for cue‐locked ERPs compared to 270 ms for feedback‐locked ERPs, and the P300 peaked at 490 ms for cues compared to 360 ms for feedback—differences that likely reflect the distinct sensory, anticipatory, and reinforcement processes engaged by each stimulus type, as well as the influence of stimulus probability on P300 latency via oddball effects. We also created a difference wave (i.e., ΔRewP) between positive and negative conditions (H1 and H2) and between cues (H1 vs. H2) to plot the topography of the reward positivity.

### 2.6 Individual Differences Analysis

To examine whether reward positivity dynamics correspond to individual differences in reinforcement learning efficiency, training phase accuracy scores from the first half of the experiment were used to classify participants into two groups (Rapid Learners and Slow Learners) using a median split. Behavioral and ERP analyses were then replicated with the inclusion of Learner group as a between‐subjects factor. In addition, Q‐learning model parameters (αGain, αLoss, and inverse temperature β) were compared between Rapid and Slow Learner groups to examine whether computational differences in reinforcement learning efficiency corresponded to the observed behavioral and electrophysiological differences. For ERP analyses, repeated measures ANOVAs on N200 amplitudes were conducted with Half (Half‐1, Half‐2) and Feedback (Positive, Negative) as within‐subject factors and Learner group (Rapid, Slow) as a between‐subjects factor. Difference waves (ΔRewP) were computed between conditions for visualization and topographic mapping purposes only.

## 3 Results

### 3.1 PST Behavioral Performance

**FIGURE 2:**
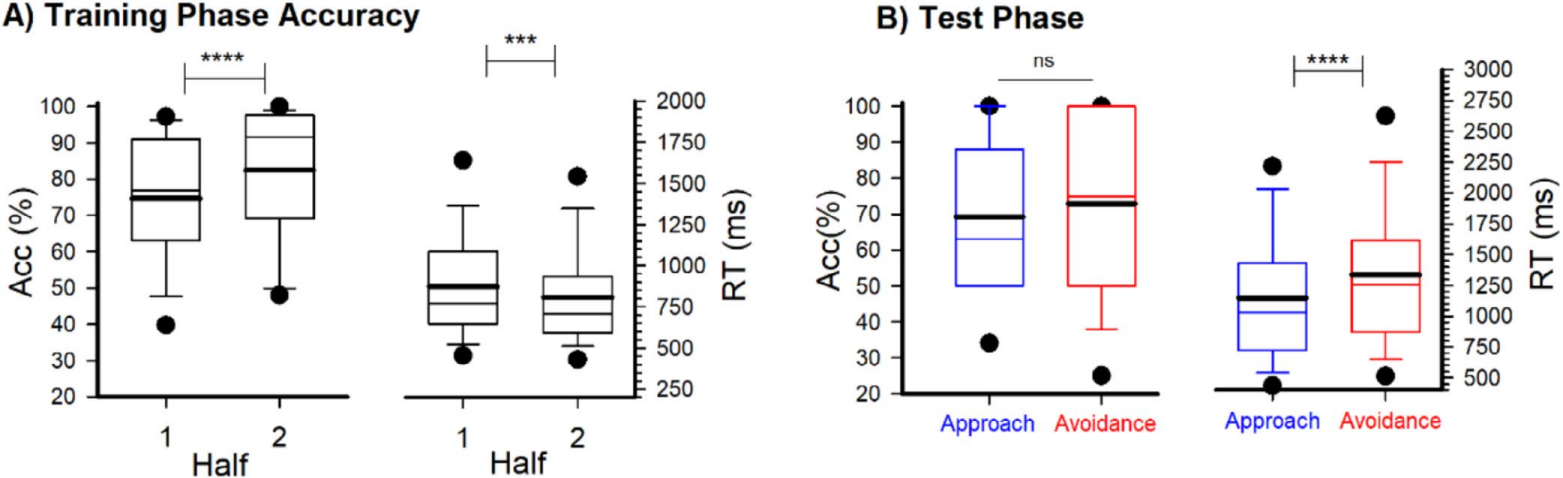
PST performance. (A) Accuracy (left panel) and reaction time, RT (right panel) across training phase halves of the PST. (B) Testing phase accuracy (% correct) and reaction time, RT (ms) for the Approach (blue) and Avoidance (red) learning. Box plots represent statistical values. The boundary of the box closest to zero indicates the 25th percentile, a thin line within the box marks the median, the thick line within the box marks the mean, and the boundary of the box farthest from zero indicates the 75th percentile. Whiskers (error bars) above and below the box indicate the 90th and 10th percentiles, and symbols denotes outlying points outside the 10th and 90th percentiles. Asterisk denotes significance of paired (within‐group) and independent (between‐group) *t*‐test results. ***p < 0.005, ****p < 0.001.

Training phase accuracy (selecting the optimal stimulus in each pair) was higher in Half‐2 (*M* = 82.5%, SEM = 2.1) compared to Half‐1 (*M* = 74.7%, SEM = 2.2), *t*(72) = −5.12, *p* < 0.001, d = 0.60, indicating that accuracy improved across the course of training (Figure 2A). Similarly, training phase reaction times were faster in Half‐2 (M = 807.5 ms, SEM = 36.4) compared to Half‐1 (*M* = 873.1 ms, SEM = 39.2), *t*(72) = 3.19, *p* = 0.002, d = 0.37 (Figure 2B). In the testing phase, accuracy did not differ between approach (*M* = 69.3%, SEM = 2.5) and avoidance (*M* = 72.9%, SEM = 3.0) conditions, *t*(72) = −0.82, *p* = 0.416, suggesting comparable learning of reward and punishment contingencies. However, participants were faster at choosing the most rewarding stimulus A (*M* = 1145.7 ms, SEM = 67.2) compared to avoiding the least rewarding stimulus B (*M* = 1335.9 ms, SEM = 75.0), *t*(72) = −3.45, *p* = 0.001, d = 0.40, suggesting faster decision‐making for approach compared to avoidance choices in the testing phase (Figure 2B). No other main effects were observed (*p* > 0.05).

### 3.2 Reward Positivity Results

**FIGURE 3:**
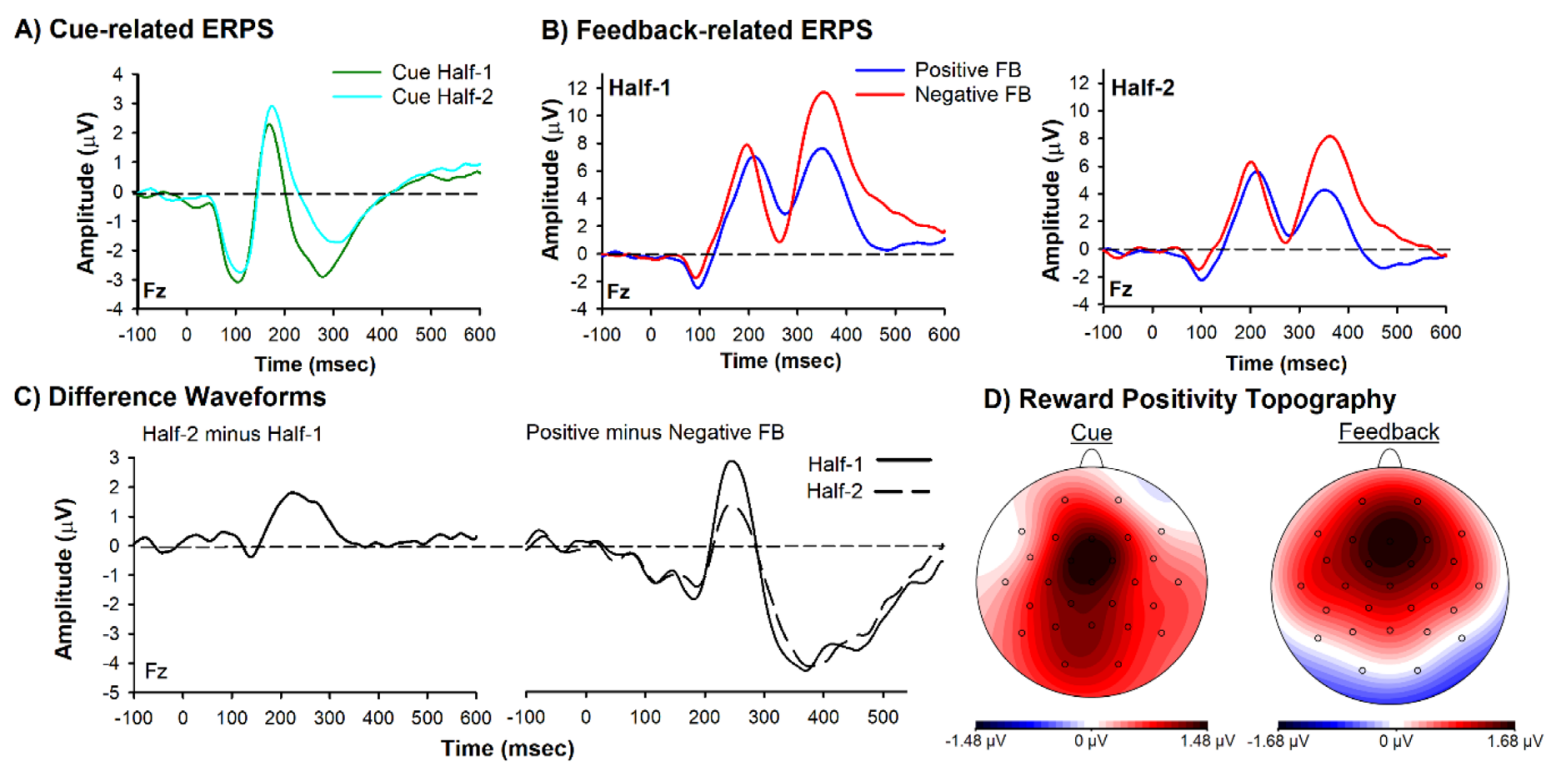
ERP results. Grand‐average ERPs recorded at channel Fz and associated difference waves and scalp distributions. (A) Grand‐average ERPs associated with Cue Half‐1(green lines) and Cue Half‐2 (cyan lines). (B) Grand‐average ERPs associated with positive (blue lines) and negative (red lines) feedback recorded at channel Fz for Half‐1 (left panel) and Half‐2 (right panel). (C) Difference waves associated with the Cue (Half‐2 minus Half‐1) and Feedback (Positive minus Negative Feedback: Half‐1[solid line] and Half‐2 [dashed line]). (D) Scalp distribution associated with the maximum of the difference wave of the Cue (Half‐2 minus Half‐1) shown in left panel and scalp distribution associated with the maximum of the difference wave of the Feedback (Half‐2 minus Half‐1) shown in right panel.

A key prediction of TD learning theory is that RPEs should shift from the time of outcome delivery to earlier predictive cues as learning progresses. Because the reward positivity and N200 overlap spatially and temporally, the reward positivity's presence or absence directly determines the observed waveform: when present, it cancels out the N200 making the waveform more positive; when absent, the N200 is exposed appearing more negative (Baker and Holroyd 2011). We therefore predicted reciprocal changes across halves. At feedback, the reward positivity to positive outcomes should decrease as rewards become expected, thereby exposing the N200 and making the waveform more negative in the second half. At cue presentation, the reward positivity should emerge as cues acquire predictive value, overlapping with the N200 and making the waveform more positive in the second half. We tested these predictions by comparing ERP amplitudes at both feedback and cue presentation across training phase halves Figure 3A,B.

A two‐way repeated measures ANOVA on N200 amplitude with Feedback (Positive vs. Negative) and Half (Half‐1, Half‐2) as factors revealed a main effect of Feedback, *F*(1, 72) = 4.563, *p* = 0.036, *η* ^2^ = 0.06, indicating that negative feedback elicited a larger N200 (mean = 1.51 μV, SEM = 0.78), compared to positive feedback, which was more positive (mean = 2.59 μV, SEM = 0.71). The main effect of Half was also significant, indicating that the N200 was more positive in the first half of the experiment (mean = 2.69 μV, SEM = 0.74) compared to the second half of the experiment (mean = 1.42 μV, SEM = 0.70). Importantly, the predicted Feedback × Half interaction was significant, *F*(1, 72) = 4.425, *p* = 0.039, *η* ^2^ = 0.06, d = 0.49. A paired‐sample *t*‐test revealed that within the first half of the experiment there was a significant difference between positive (mean = 3.45 μV, SEM = 0.75) and negative (mean = 1.92 μV, SEM = 0.83) feedback, *t*(72) = 2.81, *p* = 0.006, d = 0.23. Indeed, visual inspection of Figure 3B (left panel) shows a larger positive deflection in the ERP to positive feedback (or smaller N200) compared to negative feedback (or larger N200). By contrast, within the second half of the experiment positive feedback (mean = 1.73 μV, SEM = 0.71) and negative feedback (mean = 1.10 μV, SEM = 0.80) stimuli elicited N200s of about equal amplitude, *t*(72) = 1.14, *p* = 0.26, d = 0.10 (Figure 3B, Right panel). Given our position that variance in N200 amplitude between positive and negative feedback can result from the superposition of a positive‐going ERP component, the reward positivity, this result suggests the presence of the reward positivity during positive feedback trials in Half‐1, but absence in Half‐2 and thereby exposing the N200. It is important to note that this result was driven more by a shift in reward positivity amplitude to positive feedback between halves (ΔRewP = 1.7, SEM = 0.37; *t*(72) = 4.69, *p* < 0.001, d = 0.27), relative to negative feedback (ΔRewP = 0.8, SEM = 0.42; *t*(72) = 1.94, *p* = 0.057, d = 0.12).

Regarding the cue, a paired‐sample *t*‐test revealed that the amplitude of the N200 was more positive in Half‐2 (mean = −1.5 μV, SEM = 0.44) compared to Half‐1 (mean = −2.7, SEM = 0.44), *t*(72) = −5.33, *p* < 0.001, d = −0.31 (Figure 3A). Further, when measured as a difference wave (Figure 3C, left panel), cue‐related ΔRewP (Half‐2 minus Half‐1) and the feedback‐related ΔRewP in Half‐1 exhibited a frontal–central scalp distribution with a maximum at channel Fz (Figure 3D), a finding consistent with previous reports of the reward positivity. Together, these results show the presence of a reward positivity elicited by positive feedback in Half‐1 but not Half‐2, and the presence of a reward positivity elicited by the cue in Half‐2. This evidence demonstrates that once the subject learned the cue (and correct response) predicted the feedback outcome, the cue—rather than the feedback—elicited the reward positivity, confirming that reward positivity amplitude functions as a positive RPE signal and reflected the updating of state‐action value as predicted by TD learning theory.

### 3.3 P200 and P300

To ensure that observed effects were specific to the reward positivity and not driven by other ERP components, we examined the P200 and P300. These components are known to be sensitive to stimulus salience and probability (oddball effects) rather than RPEs, and could therefore potentially confound interpretation of reward positivity effects if not carefully controlled. In regard to the cue, while the amplitude of the P200 was also different between the second half (mean = 2.0 μV, SEM = 0.33) and the first half (mean = 1.6 μV, SEM = 0.33) of the task *t*(72) = −2.34, *p* = 0.03, d = −0.16, no differences were observed in P300 amplitude (*p* = 0.95). In regards to feedback, a two‐way repeated measures ANOVA on P200 amplitude with Feedback (Positive vs. Negative) and Half (Half‐1, Half‐2) as factors revealed a main effect of Half, F(1, 72) = 34.35, *p* < 0.001, *η* ^2^ = 0.32, indicating that the P200 was larger in the first half of the experiment (mean = 8.0 μV, SEM = 0.65) compared to the second half of the experiment (mean = 6.3 μV, SEM = 0.59). No other main effects nor interactions were observed (*p* > 0.05).

Regarding the P300, this analysis revealed a main effect of feedback, *F*(1, 72) = 41.15, *p* < 0.001, *η* ^2^ = 0.36, indicating that the P300 was larger for negative feedback (mean = 10.3 μV, SEM = 0.80) compared to positive feedback (mean = 7.3 μV, SEM = 0.69). Further, a main effect of Half was also observed, *F*(1, 72) = 58.56, *p* < 0.001, *η* ^2^ = 0.45, indicating that the P300 was larger in the first half (mean = 10.4 μV, SEM = 0.75) compared to the second half (mean = 7.2 μV, SEM = 0.73). Importantly, there was no interaction (*p* = 0.55), indicating that negative feedback elicited a larger P300 than positive feedback in the first half (negative feedback, mean = 11.8 μV, SEM = 0.83 | positive feedback, mean = 9.0 μV, SEM = 0.74, *t*(72) = −5.90, *p* < 0.001, d = 0.69) and second half (negative feedback, mean = 8.7 μV, SEM = 0.85 | positive feedback, mean = 5.7 μV, SEM = 0.70, *t*(72) = −5.54, *p* < 0.001, d = 0.65) of the experiment. Thus, the P300 findings confirm that stimulus probability (infrequent negative feedback) in the PST also elicited a P300 oddball effect, which remained stable across the training phase.

### 3.4 Individual Differences in Reinforcement Learning

**FIGURE 4:**
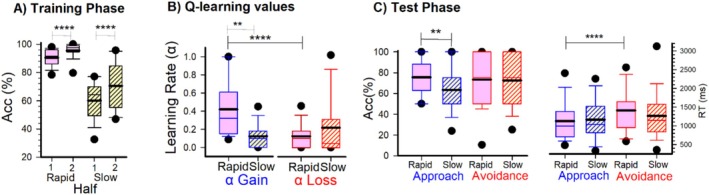
PST performance for Learner Groups. (A) Accuracy across training phase halves of the PST for Rapid (Pink Solid Boxes) and Slow (Yellow Dashed Boxes) Learners. (B) Q‐learning results. Go and no‐go learning rate values for Rapid and Slow Learner groups. (C) Testing phase accuracy (% correct) and reaction time, RT (ms) for the Approach (blue) and Avoidance (red) learning. Box plots represent statistical values. The boundary of the box closest to zero indicates the 25th percentile, a thin line within the box marks the median, the thick line within the box marks the mean, and the boundary of the box farthest from zero indicates the 75th percentile. Whiskers (error bars) above and below the box indicate the 90th and 10th percentiles, and symbols denotes outlying points outside the 10th and 90th percentiles. Asterisk denotes significance of paired (within‐group) and independent (between‐group) *t*‐test results. **p < 0.01, ****p < 0.001.

**FIGURE 5:**
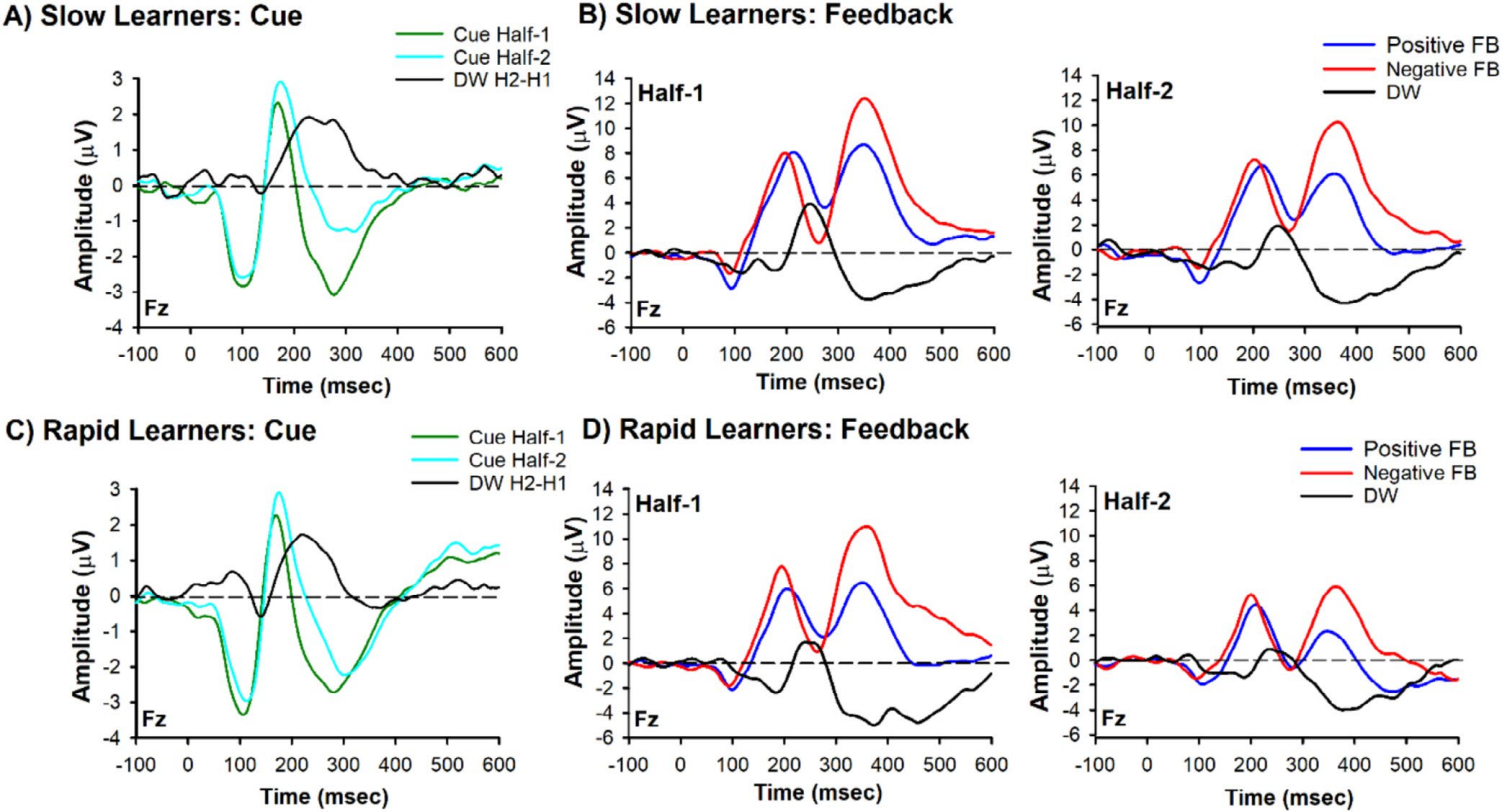
ERP results for Learner Groups. Grand‐average ERPs recorded at channel Fz and associated difference waves and scalp distributions for Rapid and Slow learners. Grand‐average ERPs associated with Cue Half‐1 (green lines) and Cue Half‐2 (cyan lines) for Slow (A) and Rapid (C) learners. Grand‐average ERPs associated with positive (blue lines) and negative (red lines) feedback recorded at channel Fz for Half‐1 (left panel) and Half‐2 (right panel) for Slow (B) and Rapid (D) Learners. Difference waves (i.e., ΔRewP) were created (Black Lines) between cues (H2 minus H1: A and C), and between positive and negative feedback conditions (H1 and H2: B and D).

If changes in reward positivity amplitude truly reflect TD learning dynamics, then individual differences in learning rate should be systematically related to individual differences in reward positivity changes. Specifically, participants who learn stimulus–reward associations more rapidly should show larger or faster shifts in reward positivity from feedback to cue between the first and second halves of the experiment. This prediction follows directly from TD theory: faster learners should accumulate prediction errors more quickly, leading to more rapid migration of the reward positivity signal backward in time. To test this prediction, training phase accuracy scores in the first half of the experiment were used to classify participants (median split) into two groups: “Rapid‐learners” (*n* = 35, accuracy scores ≥ 77 in Half‐1) and “Slow‐learners” (*n* = 38, accuracy scores ≤ 77.5 in Half‐1). We then replicated the behavioral (Figure 4A) and ERP (Figure 5) analysis above but with the inclusion of Learner group. In addition, to examine computational differences in reinforcement learning between Learner groups, we simulated PST performance using Q‐learning (Figure 4B). To minimize redundancy with the results presented above, we focused our statistical reporting on the between‐subject and interaction effects (within‐subject and between‐subject factors).

### 3.5 Behavioral Results

For training phase performance, simple effects analysis showed that both Slow Learners (Half‐1: mean = 60%, SEM = 1.7 | Half‐2: mean = 71%, SEM = 2.1, *t*(71) = 4.99, *p* < 0.001, *d* = 0.83) and Rapid Learners (Half‐1: mean = 90%, SEM = 1.8 | Half‐2: mean = 96%, SEM = 2.2, *t*(71) = 2.31, *p* = 0.024, *d* = 0.39) showed an increase in accuracy between halves (Figure 4A). Regarding Test phase performance, an ANOVA with Stimulus Condition (Approach and Avoidance) and Learner group (Rapid vs. Slow) as factors revealed a marginal main effect of Learner group, *F*(1, 71) = 3.825, *p* = 0.05, *η* ^2^ = 0.05, *d* = 0.47. No main effect of Stimulus Condition was observed (*p* = 0.445) nor an interaction *p* = 0.206. An exploratory analysis revealed that Rapid Learners performed significantly better at approach learning (Go trials: mean = 76%, SEM = 3.5) compared to Slow Learners (mean = 63%, SEM = 3.4), *t*(71) = 2.47, *p* = 0.01, *d* = 0.59, whereas no group difference were observed in avoidance learning performance (Rapid Learners: mean = 73%, SEM = 4.4 | Slow Learners: mean = 73%, SEM = 4.2, *p* = 0.901) (Figure 4C). These findings indicate that the learning differences between groups were specific to approach learning, with both groups showing comparable avoidance learning abilities. Next, an analysis of Test Phase reaction times revealed a main effect of Stimulus Condition, *F*(1, 71) = 12.725, *p* < 0.001, *η* ^2^ = 0.15, with overall slower responses on avoidance trials (mean = 1339 ms, SEM = 75) compared to approach trials (mean = 1145 ms, SEM = 68) (Figure 4C. right panel). The Feedback × Group interaction was marginal, *F*(1, 71) = 2.913, *p* = 0.092, *η* ^2^ = 0.04. Simple effects analysis showed that Rapid Learners had significantly longer reaction times on avoidance trials (mean = 1412 ms, SEM = 108) compared to approach trials (mean = 1126 ms, SEM = 98), *t*(71) = 3.66, *p* < 0.001, *d* = 0.62. In contrast, Slow Learners showed no significant difference between approach (mean = 1164 ms, SEM = 94) and avoidance trials (mean = 1265 ms, SEM = 104), *p* = 0.183. No main effect of Learner group was observed (*p* = 0.684).

### 3.6 Q‐Learning Results

In regards to learning rates, a repeated‐measures ANOVA on learning rate values, with Learning Rate (*α*_Gain_, *α*_Loss_) as a within‐subject factor and Learner Group (rapid vs. slow learners) as a between‐subjects factor, revealed a main effect of Learning Rate, *F*(1, 71) = 17.017, *p* < 0.001, $\eta_{p}^{2}$ = 0.19, indicating that learning from rewards (*α*_Gain_; *M* = 0.34, SEM = 0.03) was greater than learning from losses (*α*_Loss_; *M* = 0.17, SEM = 0.03). Interestingly, this analysis revealed a significant interaction between Learning Rate and Learner Group, *F*(1, 71) = 8.893, *p* = 0.004, $\eta_{p}^{2}$ = 0.11 (Figure 4B). This interaction indicated that Rapid Learners had significantly higher α_Gain_ values (mean = 0.42, SEM = 0.05) compared to Slow Learners (mean = 0.26, SEM = 0.05), *t*(71) = 2.41, *p* = 0.01, *d* = 0.57. By contrast, there were no significant group differences in α_Loss_ values (*p* = 0.111; see Figure 4B). Additionally, analysis of the inverse temperature parameter (β) revealed that Rapid Learners exhibited significantly higher β values (mean = 4.99, SEM = 0.01) compared to Slow Learners (mean = 4.01, SEM = 0.23), *t*(71) = 4.13, *p* < 0.001, *d* = 0.97, indicating more deterministic choice behavior (i.e., less stochastic or more consistently chose the option with the highest expected value) in the Rapid Learner group.

### 3.7 ERP Results

**FIGURE 6:**
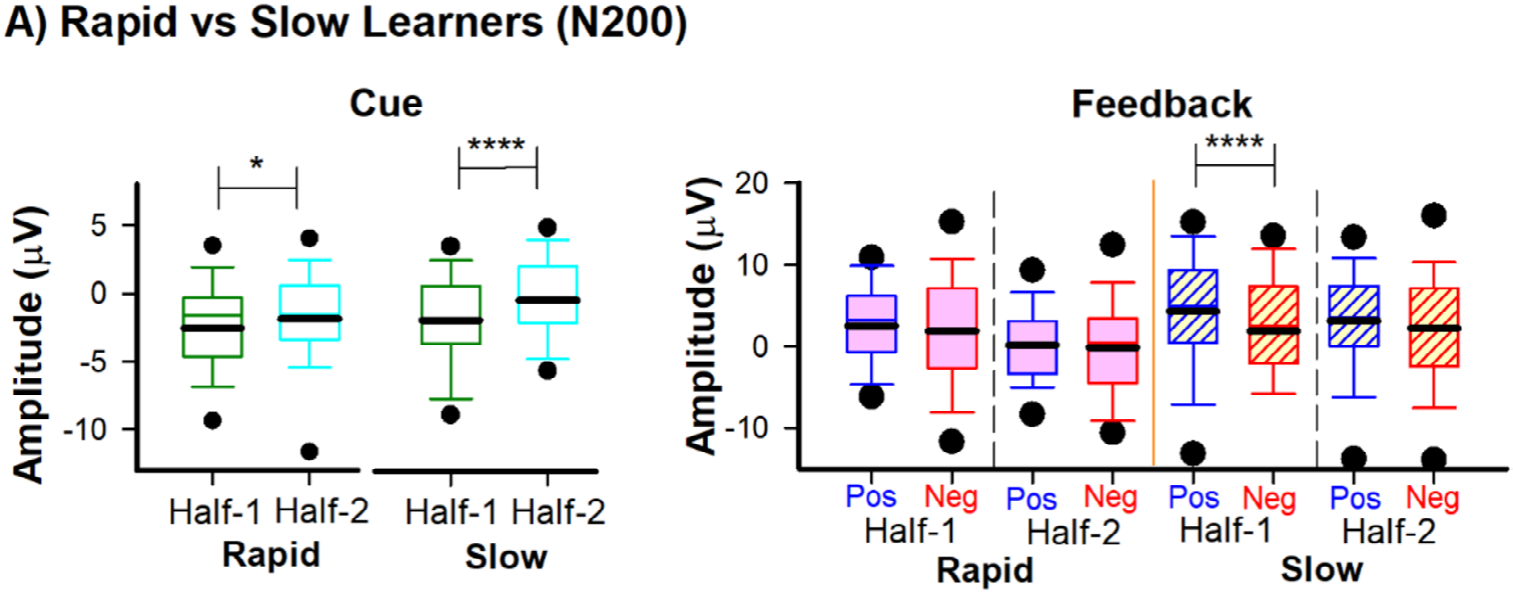
Reward Positivity and Learner Groups. (A) Reward positivity (as measured using the N200) for Half‐1 and Half‐2 for cues (left panel) and Feedback (right panel) across Rapid and Slow Learner groups. Box plots represent statistical values. The boundary of the box closest to zero indicates the 25th percentile, a thin line within the box marks the median, the thick line within the box marks the mean, and the boundary of the box farthest from zero indicates the 75th percentile. Whiskers (error bars) above and below the box indicate the 90th and 10th percentiles, and symbols denotes outlying points outside the 10th and 90th percentiles. Asterisk denotes significance of paired (within‐group) and independent (between‐group) *t*‐test results. *p < 0.05, * ****p < 0.001.

Regarding feedback, a repeated measures ANOVA on N200 amplitude with Half (Half‐1, Half‐2) and Feedback (Positive, Negative) as within‐factors, and Learner group (Rapid, Slow) as between‐factors revealed a significant Half × Learner group interaction, *F*_(1,71)_ = 7.600, *p* = 0.007, *η* ^2^ = 0.097 (Figure 5B,D). Post hoc tests indicated that there was a strong negative shift in N200 amplitude between Half‐1 (mean = 2.22 μV, SEM = 1.1) and Half‐2 (mean = 0.033 μV, SEM = 0.99) for the Rapid learners, *t* (34) = 4.62, *p* < 0.001, *d* = 0.78, but not Slow learners (Half‐1: mean =3.1μV, SEM = 1.1 | Half‐2: mean = 2.7 μV, SEM = 1.0, *t* (37) = 0.98, *p* = 0.33, *d* = 0.16. However, an exploratory analysis revealed distinct patterns between groups. The Slow Learner group differentiated between positive and negative feedback in Half‐1, *t* (37) = −6.02, *p* < 0.001, *d* = 0.48, but not in Half‐2, *t* (37) = 1.1, *p* = 0.27, *d* = 0.18, indicating a gradual propagation of the reward positivity during the training phase (Figure 6B). By contrast, the Rapid Learner group did not differ between positive and negative feedback in either Half‐1, *t* (34) = 0.87, *p* = 0.394, *d* = 0.14 or Half‐2, *t* (34) = 0.396, *p* = 0.70, *d* = 0.06, possibly indicating that reward positivity had backpropagated to the cue early in training (e.g., Block 1) (Figure 6B). No other interactions were observed (*p* > 0.05). Regarding the cue‐related ERPs, a repeated measures ANOVA on N200 amplitude with Half (Half‐1, Half‐2) and Learner group (Rapid, Slow) as factors revealed a Half × Learner group interaction, *F*_(1,61)_ = 8.56, *p* = 0.005, *η* ^2^ = 0.12 (Figure 5A,C). Post hoc tests revealed that Slow learners displayed a larger difference in N200 amplitude between Half‐1 (mean = −2.83 μV, SEM = 0.64) and Half‐2 (mean = −1.21 μV, SEM = 0.55 | ΔRewP = −1.61 μV, SEM = 0.32, *t* (37) = −6.02, *p* < 0.001, *d* = −0.97), compared to Fast learners (Half‐1: mean = −2.56 μV, SEM = 0.60 | Half‐2: mean = −1.85 μV, SEM = 0.69), *t* (34) = −2.04, *p* = 0.04, *d* = −0.35) (Figure 6A). In other words, the difference in N200 amplitude between Half‐1 and Half‐2 (i.e., ΔRewP) was larger for the Slow learners (ΔRewP = −1.78 μV, SEM = 0.32) compared to the Fast learners (ΔRewP = −0.42 μV, SEM = 0.33), *t*(61) = 2.93, *p* = 0.005.

## 4 Discussion

TD learning offers an elegant and parsimonious computational account for many (but not all) learning phenomena related to changes in cue/state values (Sutton and Barto 1998). Accordingly, phasic increases in dopamine activity occur when events are better than expected (positive RPEs) while transient cessations result when events are worse than expected (negative RPEs) (Schultz 2002). With learning, positive RPEs are hypothesized to transfer from rewarding outcomes to predictive stimuli, and are conveyed to the MCC where they are utilized to learn the value of rewards for selecting and motivating goal‐directed behavior (Holroyd and Coles 2002; Holroyd and Yeung 2012). We have argued that the impact of positive RPE signals on the MCC can be reliably measured using the reward positivity (Baker and Holroyd 2009, 2011; Holroyd et al. 2008). Supporting this claim, previous work has demonstrated that reward positivity complies with an axiomatic definition of an RPE signal, namely showing sensitivity to both the prior likelihood of reward and its magnitude on receipt (Sambrook and Goslin 2015; Walsh and Anderson 2012). Further, the component's temporal dynamics (240–340 ms) align with RPE signals recorded directly from dopamine midbrain nuclei in humans (Zaghloul et al. 2009), is modulated by genetic polymorphisms affecting prefrontal dopamine D4 receptor expression (Baker, Stockwell, et al. 2016), and is attenuated in non‐human primates by the dopamine antagonist haloperidol (Vezoli and Procyk 2009). Despite this convergent evidence, the critical prediction that reward positivity should demonstrate temporal backpropagation—shifting from feedback to predictive cues as learning progresses—has yet to be fully demonstrated. Here, we provide clear evidence of this fundamental process.

Foremost, our findings demonstrate that as participants learned stimulus‐action‐outcome associations in a probabilistic selection paradigm, the reward positivity shifted from being elicited by positive feedback early in learning to being elicited by predictive cues late in learning, consistent with computational models predicting temporal backpropagation of RPE signals as learning progresses. In more detail, early training blocks (Half‐1) showed a reward positivity elicited by positive feedback relative to negative feedback, consistent with previous literature demonstrating that unexpected positive outcomes generate positive RPE signals. This feedback‐related reward positivity diminished by the second half of training (Half‐2), when outcomes had become more predictable. It's worth noting that this reduction was primarily driven by changes in ERPs elicited by positive rather than negative feedback (i.e., positive feedback ERPs became less positive from Half‐1 to Half‐2, while negative feedback ERPs showed little change over the same period). This pattern indicates that the reward positivity component was selectively modulated by learning from positive outcomes, rather than reflecting general changes in expectancy or attention that would equally affect responses to both positive and negative outcomes. Importantly, as the reward positivity diminished with learning, it exposed the N200 on positive feedback trials, consistent with the view that the N200 is elicited by task‐relevant events in general but is suppressed by the overlapping reward positivity on trials with unexpected positive feedback (see Figure 1). This dissociation between reward positivity and the N200 has been observed in other contexts as well. For instance, increased feedback stimulus complexity can delay the onset of reward positivity, thereby exposing the underlying N200 component on both reward and no‐reward trials (Baker and Holroyd 2011). Similarly, substance dependent individuals who show impaired reward learning produce an N200 response to reward feedback that mirrors their N200 to non‐reward feedback, suggesting that reward feedback failed to elicit the expected reward positivity in this population (Baker, Stockwell, et al. 2016; Baker et al. 2011; Biernacki et al. 2020). These examples illustrate how reward positivity and N200 can be experimentally dissociated, while also demonstrating that reward positivity specifically diminishes as participants learn to predict the outcome, supporting the view that this component indexes positive RPE signals rather than reward processing in general.

While this feedback pattern was consistent with backpropagation, the critical evidence would be showing reward positivity emerging as the cues themselves acquire predictive value. Consistent with this prediction, we observed the emergence of the reward positivity to the cues in Half‐2, indicating that the predictive value had transferred from the feedback to the stimulus pairs that preceded it. Critically, this cue‐related reward positivity emerged in the N200 time window (around 270 ms), where it overlapped temporally and spatially with the N200 component making the overall ERP waveform appear more positive. As we have argued elsewhere, the reward positivity is not a discrete component but rather overlaps with the N200 as well as other neighboring ERP components (Baker and Holroyd 2011). For example, the marginal increase in cue‐related P200 amplitude likely reflects spillover effects from the emerging reward positivity component, while the much larger effects measured in the N200 time window indicate that the primary reward positivity activity occurred at the predicted latency (240–340 ms) rather than in earlier (P200) or later (P300) time ranges. To note, the amplitude of the feedback‐related P200 decreased from Half‐1 to Half‐2, consistent with the diminishing reward positivity at feedback across learning phases. Further, the topographical distribution of both cue‐related and feedback‐related reward positivity effects, as measured by the difference wave, showed the characteristic frontal‐central scalp distribution of the reward positivity. This suggests that the reward positivity elicited by both the cue and feedback likely reflected activity from similar neural generators, in particular the MCC (Baker and Holroyd 2011; Oerlemans et al. 2025). Taken together, this pattern in reward positivity amplitude supports the core prediction of TD learning algorithms that RPE signals should transfer from outcomes to predictive cues as associations are learned, and distinguish our results from previous studies that reported cue‐related ERP activity without demonstrating genuine learning‐dependent backpropagation processes (Baker and Holroyd 2009; Holroyd et al. 2011).

In computational terms, as the value function for state‐action pairs (seeing AB pair → choosing A) increased through repeated experience, the cues themselves should elicit positive RPE signals rather than the feedback that follows. This backpropagation mechanism is fundamental to how reinforcement learning systems can learn to predict rewards from increasingly distal cues and is thought to underline the development of goal‐directed behavior (Schultz 2011; Redish et al. 2008; Grace et al. 2007). By extension, our findings demonstrate that reward positivity provides a reliable electrophysiological marker for tracking this backpropagation process in real‐time, offering a direct neural measure of how predictive value transfers across temporal sequences during human reinforcement learning. Furthermore, the emergence of reward positivity at predictive cues provides support for a motivational ‘wanting’ interpretation over hedonic ‘liking’ and salience accounts of reward positivity. In particular, the hedonic interpretation is based on several recent observations that the reward positivity amplitude scales with participants' self‐reported liking of feedback stimuli and is enhanced by positive affective stimuli, even when controlling formal RPE magnitude (Brown and Cavanagh 2018; Singh et al. 2023). Others have reported that the reward positivity correlates with individual differences in reward responsiveness and extraversion, can be modulated by emotion regulation strategies, and is consistently diminished in depression—a condition characterized by reduced hedonic capacity (Proudfit 2015). To note, several of these studies employed guessing or gambling tasks rather than paradigms specifically designed to test RPE‐based learning, potentially limiting their relevance to computational accounts of RPE processing. Nevertheless, such findings have led to the proposal that the reward positivity reflects the brain's evaluation of hedonic value (i.e., indexing the subjective pleasure derived from rewarding outcomes). According to this hedonic account, reward positivity should remain anchored to the moment of reward delivery throughout learning, rather than exhibiting temporal backpropagation. In contrast to this view, the systematic temporal migration we observed—where reward positivity shifts from feedback to predictive cues as learning progresses—is incompatible with a pure hedonic account. This pattern demonstrates that the reward positivity is tied to the unpredictability of positive outcomes rather than their hedonic value, and supports the position that the reward positivity reflects MCC's role in reward valuation, which specifically drives motivational effort toward reward pursuit (“wanting”) as distinct from the hedonic enjoyment (“liking”) that occurs during reward consumption (Baker et al. 2011; Holroyd and Umemoto 2016; Baker, Wood, and Holroyd 2016).

While alternative accounts have also proposed that the reward positivity reflects a salience prediction error—responding to any motivationally significant outcome regardless of valence (Talmi et al. 2013; Brown and Cavanagh 2018)—the present findings argue against this interpretation as well. It is worth noting that the salience account maps onto the concept of an unsigned prediction error, which responds to any surprising outcome regardless of whether it is better or worse than expected, and therefore does not distinguish between positive and negative RPEs. By contrast, TD learning theory describes a signed RPE that is valence‐specific, being positive when outcomes exceed expectations and negative when they fall short. Critically, the learning‐related changes observed in the present study were largely driven by positive feedback—the reward positivity diminished at positive feedback and emerged at predictive cues across learning, while negative feedback ERPs remained relatively stable throughout. Under an unsigned salience account, both positive and negative feedback should show comparable learning‐related modulation, as both are motivationally significant outcomes. The fact that only positive feedback drove the temporal transfer is specifically consistent with a signed RPE account and directly inconsistent with an unsigned salience interpretation. The systematic temporal transfer of the reward positivity from feedback to predictive cues during learning further reinforces this conclusion, as a salience account would predict no such learning‐dependent migration—the reward positivity should remain tied to the most motivationally significant event in the sequence regardless of how much learning has occurred. Converging evidence against the salience account also comes from Heydari and Holroyd (2016), who directly tested this hypothesis by comparing monetary reward feedback to physically painful punishment feedback. Despite the punishment stimulus being rated as equally salient, it failed to elicit the reward positivity in a standard active reinforcement learning paradigm, a result directly inconsistent with the salience prediction error hypothesis and supportive of the view that the reward positivity indexes a signed, valence‐specific RPE signal (Heydari and Holroyd 2016).

Next, to examine whether RPE dynamics correspond to individual differences in learning efficiency, we divided participants into rapid and slow learner groups based on first‐half performance. This individual difference analysis revealed convergent patterns across behavioral, computational, and electrophysiological measures that illuminate the mechanisms underlying learning efficiency. Behaviorally, rapid learners achieved approximately 90% accuracy already in Block 1 and maintained this high performance throughout the training phase. In contrast, slow learners showed a more gradual improvement, requiring extended experience across all blocks to reach comparable accuracy levels. Consistent with these behavioral results, rapid learners displayed significantly higher αGain values, indicating that they made larger adjustments to their action values following positive feedback. In the Q‐learning framework, the learning rate (*α*) scales the magnitude of value updates following prediction errors—when an action yields better‐than‐expected outcomes (positive RPE), the Q‐value for that state‐action pair is updated. Higher αGain means rapid learners accumulated value estimates more quickly with each rewarding experience, creating steeper value gradients between optimal (choosing A over B) and suboptimal actions. Complementing this, rapid learners also exhibited significantly higher inverse temperature (*β*) values, indicating more deterministic choice behavior—they more consistently exploited their learned values rather than exploring alternatives. No group differences emerged in αLoss, indicating that learning efficiency differences were specific to positive feedback processing. This synergistic combination of higher αGain (faster value learning) and higher β (more consistent value exploitation) created a computational profile of enhanced reinforcement learning efficiency.

By extension, the reward positivity revealed that these computational differences had direct neural consequences for the temporal dynamics of RPE backpropagation. Slow learners showed a more pronounced shift in the cue‐related reward positivity between Half‐1 and Half‐2, suggesting a more gradual transfer of RPE signals from feedback to predictive cues as associations were acquired. At feedback, slow learners showed a differential N200 response between positive and negative feedback in Half‐1 but not Half‐2, indicating that the reward positivity gradually diminished at feedback as outcomes became expected while simultaneously emerging at cues. This pattern directly reflects the theoretical prediction that RPE signals should migrate backward in time as learning progresses. In contrast, rapid learners exhibited a different reward positivity profile. They showed a smaller change in cue‐related reward positivity between halves—not because learning was weaker, but likely because value transfer occurred earlier in training. Given that rapid learners achieved 90% accuracy within Block 1, and Half‐1 measurements averaged across Blocks 1–2, these participants had likely already completed much of the backpropagation process before the first measurement window. Supporting this interpretation, feedback‐related ERPs in rapid learners were more negative overall in Half‐2 compared to Half‐1, indicating complete absence of the reward positivity at feedback by the second half. Furthermore, rapid learners showed no differential N200 between positive and negative feedback in either learning phase, despite presumably finding positive feedback subjectively rewarding throughout the experiment. If reward positivity indexed hedonic ‘liking,’ these participants should have shown differential responses to rewarding feedback regardless of learning stage. Instead, the absence of reward positivity at feedback in rapid learners reflected that reward prediction had already been established early in training—the cues had acquired predictive value so quickly that feedback no longer generated positive RPEs.

This convergence across behavioral, computational, and electrophysiological measures demonstrates that individual differences in reinforcement learning efficiency are primarily driven by the rate of positive RPE processing. Higher αGain accelerates value accumulation at cues, causing a faster temporal transfer of the reward positivity from feedback to cue. The reward positivity changes we observed specifically reflected positive RPE backpropagation, as the reward positivity transferred from feedback to cues, with negative feedback processing remained relatively stable across groups and learning phases. The relationship between αGain and learning efficiency likely reflects underlying dopaminergic mechanisms. In TD learning theory, phasic dopamine bursts encode positive RPEs, and these signals drive synaptic plasticity in corticostriatal circuits to update action values (Cavanagh et al. 2010). Individual differences in the magnitude or reliability of these dopamine signals would be captured computationally as differences in learning rate parameters—stronger or more reliable dopamine responses to positive outcomes would manifest as higher αGain values, leading to larger value updates per trial. Consistent with this interpretation, experimental manipulations that enhance dopaminergic signaling have been shown to increase positive learning rates in the PST. For example, low‐dose D2 receptor antagonism (presumed to increase striatal dopamine availability) (Frank and Fossella 2011) and cigarette consumption (which acutely increases dopamine release) (Baker et al. 2018) both enhance learning from positive feedback and increase model‐derived αGain parameters. Similarly, repetitive transcranial magnetic stimulation (10‐Hz TMS) to the left dorsolateral prefrontal cortex—which increases dopamine release in the cingulate cortex and striatum—has been shown to selectively enhance gain learning rates relative to loss learning rates, consistent with the proposal that increasing phasic responsiveness of dopamine neurons amplifies the gain on positive RPE signals in the striatum (Biernacki et al. 2023). TMS studies using the 10‐Hz protocol have also demonstrated that potentiating dopamine activity not only enhances positive learning rates computationally but also normalizes the reward positivity in individuals with substance use disorders who characteristically exhibit blunted reward responses to non‐drug rewards (Biernacki et al. 2020, 2025).

In conclusion, this study provides strong support for the backpropagation of positive RPE signals during human reinforcement learning, as indexed by systematic changes in the amplitude of the reward positivity. The present findings extend previous work by demonstrating that naturally occurring individual differences in αGain—whether reflecting trait‐level variation in dopaminergic function, receptor density, or other neurocognitive factors (Woodward et al. 2009)—predict both the speed of behavioral learning and the temporal dynamics of neural learning signatures, as captured by the reward positivity. The temporal shift in reward positivity amplitude from feedback to cue supports core predictions of learning models and demonstrates that the MCC implements such mechanisms for learning predictive associations. Individual differences in these neural dynamics appear to be related to learning efficiency, with implications for understanding both normal variation and potential clinical applications. For example, conditions such as depression, substance use disorder, and schizophrenia have been linked to altered dopaminergic function and reinforcement learning deficits, and understanding how the temporal dynamics of reward positivity signals differ in these populations could provide key insights into the neural mechanisms underlying these disorders. In sum, these findings advance our understanding of the neural mechanisms underlying human reinforcement learning and provide a foundation for future reward positivity investigations into the dynamic nature of RPE‐guided learning in the brain.

### 4.1 Future Directions and Clinical Implications

The reward positivity findings reported here have important implications for both ERP methodology and clinical research. Previous EEG studies using probabilistic learning paradigms have focused on comparing positive versus negative feedback without considering how these responses change as learning progresses, often averaging ERPs across the entire experiment. This averaging approach misses crucial dynamics in the learning process and may conflate different stages of learning with different ERP components signatures (Krigolson 2018). For example, in contrast to the reward positivity findings, the P300 component remained stable across learning phases, with P300 being consistently larger for negative compared to positive feedback in both Half‐1 and Half‐2, and no significant interaction between feedback type and learning phase. This stable difference between positive and negative feedback throughout the experiment indicates that the P300 likely reflected stimulus probability effects rather than learning‐specific changes (e.g., negative feedback occurred on 20%–40% of trials, making it less frequent than positive feedback). The stable and larger P300 to negative feedback across both learning phases is consistent with the well‐established oddball effect, where the P300 (and in some cases the N200) is enhanced for infrequent or unexpected stimuli regardless of their valence or learned significance (Hajihosseini and Holroyd 2013; Holroyd et al. 2008). Furthermore, while positive RPEs clearly back‐propagate (reward positivity changes over learning), negative feedback ERPs didn't show comparable modulation. This raises a fundamental question: Do negative RPEs drive cue‐based learning at all, or do they serve a different computational role, such as triggering immediate behavioral adaptations like post‐error slowing? Alternatively, the current PST paradigm or ERP measures may be insensitive to negative RPE back‐propagation, thus warranting future investigations.

These methodological considerations also have important implications for clinical research, where ERP measures are often related to mental health conditions like depression, anxiety, and substance use disorders. Clinical studies commonly measure feedback‐related components using wide time windows (e.g., mean amplitude 200–450 ms) or base‐to‐peak measures (P200‐P300 or N200‐P300) that capture overlapping processes from distinct neural systems: reward system (reward positivity), cognitive control system (N200), and attention/arousal system (P300) (Krigolson 2018). In probabilistic learning tasks where negative feedback is typically less frequent, these measurement approaches confound valence with frequency effects, making it impossible to determine which system drives the observed clinical differences (Hajihosseini and Holroyd 2013; Krigolson 2018). Consider measuring the P3‐FRN (the amplitude difference between P300 and N200) during probabilistic learning. One study found that anxiety strongly correlated with the degree to which an individual's negative RPE was reflected in the punishment‐locked P3‐FRN component—meaning more anxious individuals showed stronger neural encoding of unexpected negative outcomes (Cavanagh et al. 2019). Another study found that depressed individuals showed elevated P3‐FRN (but not P2‐FRN measure) compared to controls, interpreted as hypersensitivity to negative outcomes (Cavanagh et al. 2011). Because P3‐FRN is computed as a difference (P300—N200), the same elevated score could arise from enhanced P300, enhanced N200, or both, and thus difficult to determine which underlying neural process drives the anxiety and depression findings. To add to this ambiguity, while a meta‐analyses of anxiety show consistent N200/FRN/FMT enhancement reflecting altered cognitive control (Cavanagh and Shackman 2015), anxiety disorders are strongly associated with norepinephrine dysregulation (Bandelow et al. 2016, 2017)—the system that modulates P300 responses to motivationally relevant stimuli (de Rover et al. 2015; Nieuwenhuis et al. 2005, 2011). However, P300 responses in anxiety vary considerably: increased to unpredictable or salient stimuli, decreased in panic disorder or OCD, or unchanged depending on context (Zhu et al. 2024). This interpretive challenge is further complicated by evidence suggesting the “oddball N2” itself may reflect noradrenergic modulation of task‐relevant cortical areas (Warren et al. 2011), potentially sharing neurobiological mechanisms with P300 (i.e., the N2‐P3 complex) rather than representing a distinct cognitive control signal. Without methodological precision to dissociate these distinct neural systems—including narrower measurement windows and experimental designs that decouple valence from frequency (e.g., reward tasks with equal outcome probabilities)—clinical findings risk misattributing ERP differences and potentially misdirecting treatments.

Additionally, the paucity of studies examining cue‐related ERPs has meant that the backpropagation predictions of TD learning theory have gone largely untested and may be more sensitive to mesocorticolimbic dysregulation than feedback‐related ERPs. This has important implications for understanding both normal variation in learning, development, and studying psychiatric conditions. For example, the differential patterns of reward positivity changes between rapid and slow learners suggest that individual differences in learning efficiency are reflected in distinct patterns of neural dynamics during reinforcement learning. Thus, without considering temporal dynamics, a diminished reward positivity in one group could reflect either faster learning or impaired reward processing, while heightened reward positivity could reflect either slower learning or enhanced reward sensitivity. Regardless of whether these components are measured in the time domain or frequency domain, future research needs to dissociate these components from overlapping cognitive constructs such as attention, stimulus frequency processing, and general expectancy violations. Only through such methodological precision can the field achieve reliable measures of reinforcement learning mechanisms that advance both theoretical understanding and clinical applications.

## Author Contributions

**Robert Wilson:** writing – review and editing. **Yifan Gao:** writing – original draft, methodology, writing – review and editing, formal analysis, data curation, investigation. **Travis E. Baker:** conceptualization, investigation, funding acquisition, writing – original draft, methodology, formal analysis, project administration, visualization, data curation, supervision. **Galit Karpov:** methodology, writing – review and editing, formal analysis, data curation, investigation.

## Funding

This work was supported by Rutgers Start‐up funds for T.E.B., and Rutgers Center for Alcohol Studies seed grant awarded to T.E.B.

## Conflicts of Interest

The authors declare no conflicts of interest.

## Data Availability

The data that support the findings of this study are available on request from the corresponding author. The data are not publicly available due to privacy or ethical restrictions.

## Endnotes

^1^
Component overlap refers to the fact that ERP components inherently overlap in time and space, such that voltage changes at the scalp reflect the summation of multiple underlying neural processes. In the case of the N200 (negative deflection) and reward positivity (positive deflection), these components are generated simultaneously following positive feedback. Because they have opposing polarities, their electrical fields summate at frontal‐central scalp electrodes and partially cancel each other out, causing the N200 to appear smaller or absent on reward trials compared to loss trials. Consequently, the fERN—historically defined as the larger negative deflection in the N200 to negative compared to positive feedback—is therefore primarily driven by the presence of the reward positivity on positive feedback trials rather than by an enhanced negative deflection to negative feedback per se.

^2^
In the standard PST, participants advance to the testing phase once they satisfy a performance criterion better than chance (65% A in AB, 60% C in CD), or after six blocks (360 trials) if not met. This design transfers participants to the testing phase as soon as the cue‐action‐outcome contingency is learned, leaving few or no training trials after learning has occurred—precisely when the ERP signatures of RPE backpropagation would be expected to emerge. The resulting variability in trial counts across participants and lack of post‐learning trials are therefore not optimal for an ERP analysis of RPE backpropagation between feedback and cue.
